# Genome-Wide Identification of *NRT* Gene Family and Expression Analysis of Nitrate Transporters in Response to Salt Stress in *Poncirus trifoliata*

**DOI:** 10.3390/genes13071115

**Published:** 2022-06-22

**Authors:** Zeqi Zhao, Mengdi Li, Weiwei Xu, Ji-Hong Liu, Chunlong Li

**Affiliations:** 1 Key Laboratory of Horticultural Plant Biology (MOE), College of Horticulture and Forestry Science, Huazhong Agricultural University, Wuhan 430070, China; zzq13296520023@163.com (Z.Z.); 18985662535@163.com (M.L.); wei_wei_xu@126.com (W.X.); liujihong@mail.hzau.edu.cn (J.-H.L.); 2 Hubei Hongshan Laboratory, Wuhan 430070, China

**Keywords:** nitrate transporter, genome-wide identification, salt stress, gene expression, *Poncirus trifoliata*

## Abstract

The uptake and transportation of nitrate play a crucial role in plant growth and development. These processes mostly depend on nitrate transporters (NRT), which guarantee the supplement of nutrition in the plant. In this study, genes encoding *NRT* with Major Facilitator Superfamily (MFS) domain were identified in trifoliate orange (*Poncirus trifoliata* (L.) Raf.). Totally, 56 *NRT1s*, 6 *NRT2s*, and 2 *NAR2s* were explored. The bioinformation analysis, including protein characteristics, conserved domain, motif, phylogenetic relationship, *cis*-acting element, and synteny correlation, indicated the evolutionary conservation and functional diversity of *NRT* genes. Additionally, expression profiles of *PtrNRTs* in different tissues demonstrated that *NRT* genes possessed spatio-temporal expression specificity. Further, the salt condition was certified to induce the expression of some *NRT* members, like *PtrNPF2.1*, *PtrNPF7.4*, and *PtrNAR2.1*, proposing the potential role of these NRTs in salt stress response. The identification of *NRT* genes and the expression pattern analysis in various tissues and salt stress lay a foundation for future research between nitrogen transport and salt resistance in *P. trifoliata*.

## 1. Introduction

Nitrogen (N) is a macro element for plant growth and development [1]. On the one hand, it is an important basic component for bio-macromolecules, like polypeptide, nucleic acid, and chlorophyll, which are essential for physiological activities in plants such as photosynthesis and protein synthesis. On the other hand, nitrogen metabolites are crucial substances that influence an organism’s development and differentiation. The sufficient supplement of nitrogen is directly related to the leaf color and shape and increases the capacity of light utilization, thus resulting in the high biological activity of plants [2,3]. In addition, nitrogen absorption and assimilation affect protein synthesis. Nitrate ion (NO_3_^−^) and ammonium ion (NH_4_^+^) are two primary inorganic forms of nitrogen in soil [4]. During the nitrogen assimilation process, NO_3_^−^ is transformed to NH_4_^+^ through the primary nitrate assimilation pathway, which is performed by the core enzymes, nitrate reductase (NR), and nitrite reductase (NiR). In plants, nitrogen is synthesized into amino acids through the glutamine synthetase/glutamate synthase (GS/GOGAT) pathway for protein synthesis [2,5]. Given the vital role of N, the level of nitrogen use efficiency (NUE) is considered a key character for crops. NUE is dependent on the process of nitrogen absorption and transportation. For that, nitrogen has to be absorbed efficiently and transported rationally, which are mainly processed by the membrane-localized transporters.

NRTs are a group of NO_3_^−^ ion transporters and function in nitrate absorption and transportation in plants. There are three main sub-families of *NRT* members, including nitrate transporter 1/peptide transporter (*NRT1/PTR*, also named *NPF*), nitrate transporter 2 (*NRT2*), and nitrate transporter 3 (*NRT3/NAR2*). According to the functional difference, the *NRTs* are defined as low-affinity transport system (LATS, including *NPFs*) and high-affinity transport system (HATS, including *NRT2s* and *NAR2s*) [6]. Homologous genes of *NRT* can be found in almost all higher plants. A total of 125 and 39 *NRT* genes were respectively identified in soybean (*Glycine soja*) [7] and potato (*Solanum tuberosum*) [8]. Due to the high degree of convergence, the peptide transporters (PTRs) are considered to be the same family as NRT1. Therefore, the NRT1/PTR family is also recognized as *NPF*, which is the largest sub-family of nitrate transporters [9]. In the *Arabidopsis thaliana*, 53 *NPF* members were identified and divided into eight sub-clades, including *NPF1*-*NPF8* [10]. Besides, 57, 68, and 73 *NPF*s were respectively identified in spinach, poplar, and apple [11,12,13]. Moreover, the *NRT2* family is the high-affinity transport system (HATS), which is mainly induced by the lower concentration of nitrate conditions in the soil. Previous research revealed that there are seven *NRT2* members in *A. thaliana*, named *AtNRT2.1* to *AtNRT2.7* [14]. For *NRT3*, it was reported to interact with the *NRT2* in protein level and finally enhance the activity of HATS [15]. So, *NRT3* was also recognized as nitrate assimilation-related gene 2 (*NAR2*). Based on the previous reports, *NRT* family members play diverse roles in the absorption and transportation of nitrogenous substances according to their specific tissue expression patterns. For instance, *AtNRT1.5* is mainly expressed at the root pericycle cells for xylem nitrate ions loading to complete the long-distance transport of nitrogen [16]. AtNRT1.9 promotes the entrance of nitrate into root phloem and facilitates nitrate down-transport in roots [17]. AtNRT1.11 and AtNRT1.12 were reported to regulate the redistribution of nitrate content in xylem and phloem [18]. Moreover, much evidence indicates that NPFs possessed the potential activity of low-affinity nitrate transportation in many species, such as *Brassica campestris*, chrysanthemum, and rice [19,20,21]. As for *NRT2s*, *AtNRT2.1* and *AtNRT2.2* were mainly expressed in the epidermis of roots for nitrate absorption [22,23]. AtNRT2.4 was located on the plasma membrane of root epidermal cells and acted as a supplementary protein for AtNRT2.1 and AtNRT2.2. Consistently, the nitrate absorption was dramatically inhibited in *atnrt2.1/2.2/2.4* triple mutation plants [24]. *AtNRT2.5* is mainly expressed in the epidermis and the cortex of the root hair zone and takes part in nitrate loading into the phloem during nitrate remobilization [25]. *AtNRT2.6* is highly expressed in vegetative organs and responds to biotic and abiotic stresses [25]. In addition, the AtNRT2.7 protein is located on the seed vacuolar membrane and plays a potential role in the nitrate accumulation at the later development stage of seeds [26]. The homologous genes of *NRT2* in other plants like cassava, *Brassica rapa*, cucumber, and chrysanthemum are also identified as high-affinity nitrate transporters with the function of nitrate transportation [27,28,29,30]. The NAR2s (NRT3s) are proven to play a significant role in nitrate transport by regulating the activity of high-affinity transport system proteins. It was reported that the AtNAR2 interacted with all of the AtNRT2 proteins except for AtNRT2.7 in *A. thaliana* [31]. The interaction of NRT2 and NAR2 was demonstrated to enhance the nitrate transport activity of a high-affinity transport system [11,15].

As an important nutrient element, nitrogen has been reported in many in-depth studies of plant salt stress response. A gradually gaining acceptance is that the processes of nitrogen assimilation, metabolism, and transportation are in profound connection with plant salt stress response. Salt stress is the principal abiotic stress for a plant. It has been reported that one-fifth of cultivated land is suffering soil salinization over the world. Increasing soil stress affects plant growth and threatens food security [32]. Under the salt condition, the enhancement of osmotic stress will lead to the decrease in available water in plants. Moreover, the accumulation of special ions will cause cellular toxicity and impair the balance of element absorption, especially for those of microelements [33]. Several scientific studies supposed that nitrogen could affect ion absorption under salt stress. Studies on sorghum and tomato found that the provision of exogenous nitrogen could effectively alleviate the absorption of Na^+^ and increase the content of K^+^ in plants [34,35]. The research on mustard showed that exogenous nitrogen could reduce the absorption of Na^+^ and Cl^−^ under salt stress [36]. Nitrate, as the substitution of negative ion of Cl^−^, was proposed to be collected to alleviate the excess chloride toxicity in plants [37]. Accordingly, nitrate was reported to be more suitable for plant growth under salt conditions than ammonium nitrogen [38]. Further, the expression of *NR* and *NiR* genes were reported to be significantly increased in *Zostera marina* under salt stress [39]. The up-regulated *NR* gene was involved in maintaining cell ion homeostasis [40]. All of these researches supported that nitrogen played a potential role in assisting plants in overcoming salt stress. However, the function of NRTs in plant salt stress response is still unclear so far.

Citrus is a widely planted fruit tree over the world. Due to the vegetative propagation, the nutrition absorption and stress tolerance of citrus are largely dependent on the grafting rootstock. Trifoliate orange is a universal rootstock for citrus culture. However, some studies figured out that trifoliate orange is more sensitive to salt stress [41,42], which limited citrus production. Nevertheless, excessive fertilizer use and the subsequent deepening soil salinization make increasingly harsh cultivation conditions for citrus and threaten fruit yield. Given the fact that *NRT* genes in *P. trifoliata* have not been identified yet, the potential relationship between nitrate uptake and salt stress response remains to be revealed in citrus. In the present work, we identified 64 *NRTs* in *P. trifoliata* through the Hidden Markov Model (HMM). The analysis of protein characteristics, conserved domains, motifs, phylogenetic relationship, *cis*-acting elements, and synteny correlation were performed to show the conservation and relationship between the orthologous and paralogous genes. In consideration of the connection between nitrogen and salt stress, we also detected the expression of *NRT* genes in different tissues and in response to salt conditions (200 mM NaCl) via RNA-seq and qRT-PCR assay. This work could be the first step for further exploring the function of NRTs both in nitrogen uptake and salt stress response in *P. trifoliata*.

## 2. Materials and Methods

### 2.1. Plant Material and Treatment

Trifoliata orange seeds were harvested from the Centre of Citrus Plant at Huazhong Agriculture University. The seeds were washed clean with water and stored at 4 °C refrigerators. All the seeds were stepwise stirred 7 min with the 1 M NaOH and another 7 min with 5% NaClO to remove pectin and inhibit bacteria. The seed germination process was carried out at a constant temperature growth chamber in dark conditions at 25 °C. The seedlings were grown in hydroponic conditions at 23 °C with the nutrition medium. The medium formula was presented in Table S1A. The salt treatment was performed on two-month-old seedlings with 200 mM NaCl at the indicated time (6 days). Low nitrogen treatment was designed to reduce the nitrogen content (NO_3_^−^) from 5 mM to 0.1 mM (Table S1B).

### 2.2. Identification of NRTs in P. trifoliata and Amino Acid Characteristic Prediction

To identify nitrate transporter genes in *P. trifoliata*, Citrus Pan-genome to Breeding Database (CPBD, http://citrus.hzau.edu.cn/index.php, accessed on 1 March 2022) was used to obtain the gene sequences and gene annotations. The Hidden Markov Model (HMM) profiles of PTR2 (PF00854), MSF_1 (PF07690), and NAR2 (PF16974) were downloaded from Pfam database (http://pfam.xfam.org/, accessed on 1 March 2022), which represented *NRT1/PTR*, *NRT2*, and *NAR2* respectively. HMM search tool was applied to identify possible NRT members from the protein sequence database of *P. trifoliata* by TBtools (Toolbox for Biologists v1.09857) with an E-value cutoff of 1.0 × 10^−5^ [43]. Then, all the hypothetical protein sequences were submitted to the National Center for Biotechnology Information (NCBI, https://www.ncbi.nlm.nih.gov/Structure/bwrpsb/bwrpsb.cgi, accessed on 1 March 2022) to verify the constitute of conserved domains by the batch CD-search tool. To reveal the character of the NRT protein sequences, the physiochemical data was calculated by the ProtParam tool of the EXPASY program (https://web.expasy.org/protparam/, accessed on 1 March 2022). The online tool was employed to detect the number of amino acids, molecular weight, theoretical isoelectric point, and grand average of hydropathicity (GRAVY) [44].

### 2.3. Phylogenetic Tree Construction and Synteny Correlation Analysis

To identify the homology of *NRT* genes between intraspecific and interspecific, a phylogenetic tree was constructed with the *A. thaliana* and *P. trifoliata* NRT protein sequences. The members of nitrate transporter in *A. thaliana* were downloaded from The Arabidopsis Information Resource (TAIR, https://www.arabidopsis.org/, accessed on 2 March 2022). Peptide sequences of *NRT* genes from *A. thaliana* and *P. trifoliata* were put into MEGAX64 software [45]. Sequences were aligned by ClustalW with the default parameters; then the phylogenetic tree was constructed by the Maximum Likelihood (ML) method. Finally, the high-quality figure of the phylogenetic tree was made through the ITOL (https://itol.embl.de, accessed on 2 March 2022). According to the structure of the tree and the real position with *A. thaliana*, 64 *PtrNRT* genes were initially named. To observe the tandem and segmental duplication of *P. trifoliata NRT* genes, the collinear correlation analysis was carried through the MCScanX by offering the gene annotation and the whole genome sequence of *P. trifoliata* and *A. thaliana*. The circos plot was constructed by Tbtools software. The synteny of interspecific and intraspecific were merged together. 

### 2.4. Gene Structure and cis-Acting Elements Analysis

To further identify the gene structure, MEME (https://meme-suite.org/meme/, accessed on 2 March 2022) was used to find out conserved nucleotide motifs (the maximum numbers of motifs were set up to 10) with default parameters. The data was obtained as the result of a batch CD-search of NCBI. The conserved domains were rechecked by SMART (http://smart.embl-heidelberg.de/smart/batch.pl, accessed on 9 May 2022) and visualized. A gene structure annotation file was downloaded to extract *NRT* gene information by accession number, which was called in visualizing the length of UTR and CDS. Furthermore, the upstream 2000 nucleotide promoter sequences of *PtrNRT* were extracted from the genome file. The PlantCare (http://bioinformatics.psb.ugent.be/webtools/plantcare/html/, accessed on 2 March 2022) was used to detect the promoter’s *cis*-acting elements [46,47]. 

### 2.5. RNA Extract, cDNA Reverse Transcription, and Gene Expression Analysis via qRT-PCR

The collected *P. trifoliata* seedlings and tissues were ground into a fine powder with liquid nitrogen for RNA extraction, which was performed according to the instruction manual using the OminiPlant RNA Kit (Cowin BIO. Wuhan, China). The Easy Script ^®^One-Step gDNA Removal and cDNA Synthesis Super Mix (Trans Gen Biotech Co., Ltd., Beijing, China) were applied to reverse-transcribe RNA into cDNA. Then, the qRT-PCR experiment was carried out with the Applied biosystem^®^QuantStudio^TM^ 7 Flex Real-Time PCR System (ABI, Los Angeles, CA, USA). The gene-specific primers were designed from NCBI, and all primers were listed in Table S2. The *actin* gene was selected as the reference. The relative expression profile was calculated by the 2^−∆∆CT^ method. The control group and the root tissue were set as 1 against the NaCl, low nitrogen groups, and different tissues, respectively. Gene expression analyses were repeated at least three times. The significant difference was analyzed by using Student’s *t*-test. The error bar indicated the standard deviation (SD). 

### 2.6. RNA-seq Data Analysis

RNA-seq was performed by the Illumina platform (carried by Novogene Technology Co., Ltd., Nanjing, China). The raw data was processed using Hisat2 and Stringtie plugins from TBtools. The differential expression analysis of *PtrNRT* genes was performed using the DESeq2 R package (1.20.0). DESeq2 provides statistical routines to determine differential expression in digital gene expression data via using a model based on the negative binomial distribution. The resulting *p*-values were adjusted using Benjamini and Hochberg’s method to control the false discovery rate.

### 2.7. Sequence Annotation, GO, and KEGG Enrichment Analysis

Protein sequences were uploaded on the eggNOG-Mapper (http://eggnog-mapper.embl.de/, accessed on 10 April 2022) to get the GO and KEGG background annotation files. The genes that log2 Fold Change absolute values greater than 1 were employed to perform GO and KEGG enrichment by TBtools. The cut-off of the *p*-value was set at 0.05 and then dealt with Benjamini and Hochberg’s approach for bubble plot.

## 3. Result

### 3.1. Genome-Wide Identification of NRT Genes in P. trifoliata

According to the previous reports, *NRT* genes were divided into three sub-families, *NPF*, *NRT2*, and *NAR2*. To identify the *NRT* members in *P. trifoliata*, protein sequences of NPF, NRT2, and NAR2 in *A. thaliana* were employed to perform Hidden Markov Model (HMM) scan. Core domains containing PTR2 (PF00854), MFS_1 (PF07690), and NAR2 (PF16974) were identified, respectively, through HMM scan. Then, HMM search in the *P. trifoliata* genome was carried out with the *e*-value cutoff in 1.0 × 10^−5^ by TBtools. Only the peptide sequences that shared the same conserved domains with *A. thaliana* were identified as a member of *PtrNRTs*. Finally, 64 *NRT* genes were selected, including 56 *NPFs*, 6 *NRT2s*, and 2 *NAR2s* in *P. trifoliata* (Table 1), which was similar to the number of other species in previous studies [10,11,12,13]. For further analysis, the peptide sequences, annotation files, and CDS sequences of *PtrNRT* genes were acquired from the CPBD. The protein sequence characteristics, including the number of amino acids (AA), start and end site, molecular weight, theoretical isoelectric point, and grand average of hydropathicity, were presented in Table 1. Basically, the AA length of NPFs and NRT2s was in a rational range from 450 to 600. The longest AA sequence was 895 (PtrNPF4.1), while the shortest one was 419 (PtrNRT2.5). Accordingly, the molecular weight of PtrNRTs ranged from 45.27 (PtrNRT2.5) to 98.89 (PtrNPF4.1) kDa. However, NAR2 was a special group with a short sequence length and small molecular weight. With respect to the theoretical isoelectric point, most of the NRT proteins preferred alkaline, but still, a few proteins were supposed to be acidic, including PtrNPF5.12, PtrNAR2.2, PtrNPF8.2, PtrNPF3.1, PtrNPF7.3, PtrNPF8.3, PtrNPF6.5, and PtrNPF5.7. Besides, only the PtrNAR2.1 possesses the negative GRAVY value, implying it might be exposed to the hydrophilic zone to perform a special function.

### 3.2. Phylogenetic Relationship and Synteny Correlation Analysis

**Figure 1:**
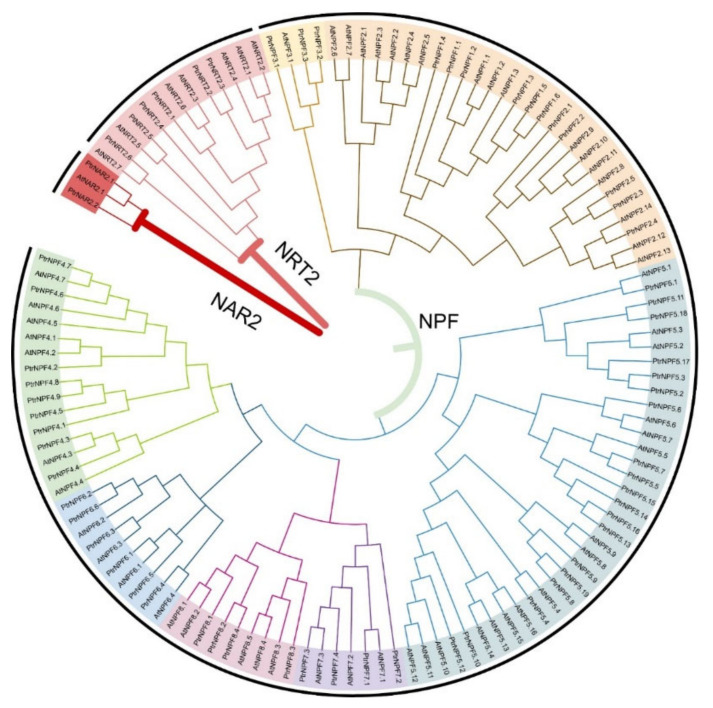
Phylogenetic analysis of NRT members from *P. trifoliata* and *A. thaliana*. A total of 64 NRT protein sequences from *P. trifoliata* (56 *NPFs*, 6 *NRT2s*, and 2 *NAR2s*) and 61 from *A. thaliana* (53 *NPFs*, 7 *NRT2s*, and 1 *NAR2*) were aligned by ClustalW method. The MEGAX program was applied to construct the phylogenetic tree by the ML method in the default parameters. The whole tree was divided into three parts, NRT1/PTR, NRT2, and NAR2. The modification of the tree was carried out on the iTOL (https://itol.embl.de/, accessed on 1 March 2022).

**Figure 2:**
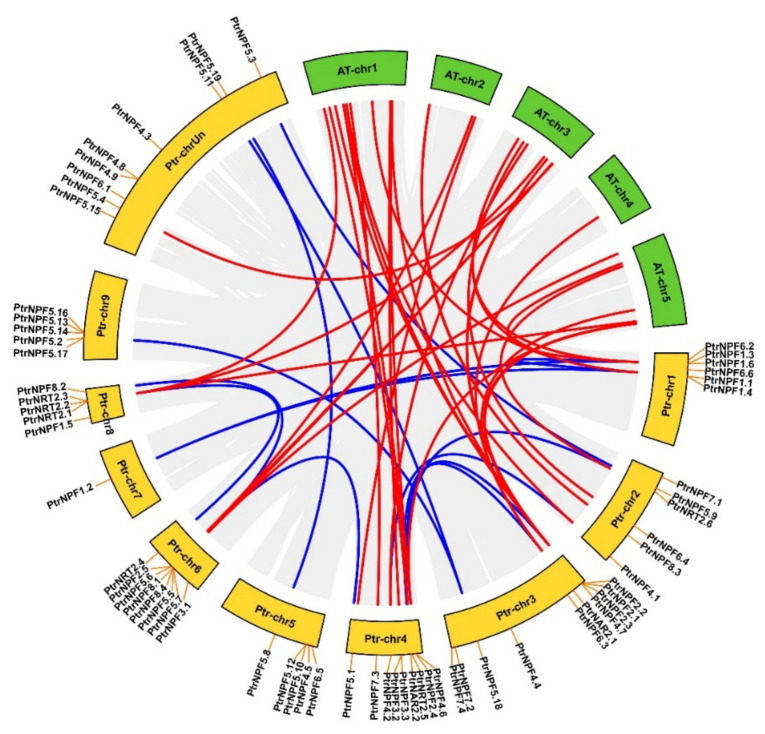
Analysis of evolutionary relationship and gene location of *NRT* family members in *P. trifoliata*. The red lines were the syntenic gene pairs among *A. thaliana* and *P. trifoliata* genomes. The blue lines were the syntenic gene pairs of *P. trifoliata*. Chr referred to chromosome. The location of *NRT* genes on chromosomes were labeled.

To reveal the homologous relationship between the NRT members, MEGAX64 was applied for the alignment assay with the peptide sequences of *P. trifoliata* and *A. thaliana* by the ClustalW under the default parameters. The phylogenetic tree was constructed for re-naming reference through the maximum likelihood (ML) method. It was shown that NRT members were grouped into three sub-families, including NPF, NRT2, and NAR2 (Figure 1). Accordingly, NPF sub-family contained most of the NRT members, while the NAR2 sub-family had only 2 and 1 members in *P. trifoliata* and *A. thaliana,* respectively. Besides, NPF sub-family could be classified into seven groups in *P. trifoliata*. Among them, PtrNPF5 had the most of 19 members, from PtrNPF5.1 to PtrNPF5.19. To further study the synteny and collinearity relationship of *NRT* genes, the circos plot was constructed by TBtools according to the gene structure annotation file (Figure 2). This plot showed the location and the evolutionary relationship of *PtrNRT* members on the chromosome level. For the *NPF* sub-family members, they were unevenly distributed on all nine chromosomes of *P. trifoliata*. Based on the statistical data, chromosome 7 contained only one family member of *PtrNPF1.2.* In addition, chromosome 3 possessed 10 *NRT* genes, which was the most among all chromosomes. *NRT2* sub-family genes were located at chromosomes 2, 4, 6, and 8. Interestingly, chromosome 8 showed a relative gathering trend, containing half of the *NRT2* genes. Besides, two *NAR2* genes were located at chromosomes 3 and 4, respectively. The distribution of *NRT* members suggested the diversity of *PtrNRT* genes in the process of evolution. The lines on the circos plot presented the gene pairs with collinearity. Despite a distant evolutionary relationship, 39 out of 51 gene pairs were still referred to as the orthologous genes (red lines) between *A. thaliana* and *P. trifoliata*, indicating that the frequency of *NRT* family amplification events is low and the members possess a relative conservative property in the process of evolutionary history.

### 3.3. Motifs, Gene Structure, and cis-Acting Elements Analysis

**Figure 3:**
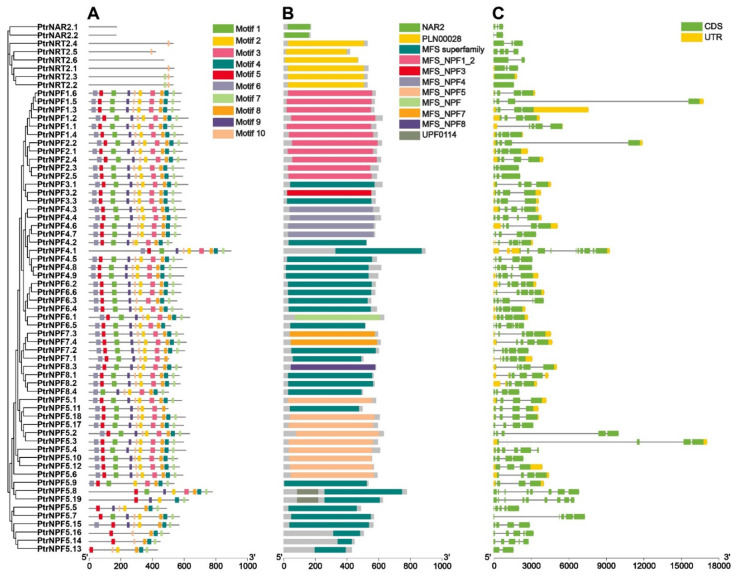
Motif, conserved domain, and gene structure analysis of *PtrNRT* genes. (**A**) The motif analysis was performed on the MEME (https://meme-suite.org/meme/doc/meme.html, accessed on 1 March 2022). Ten motifs were identified. The detail of motif sequence information was presented in supplemental Figure S1. (**B**) The conserved domain analysis was performed through the NCBI Batch CD-search tool. (**C**) The gene structure analysis. The yellow color was the untranslated region, while the green was the coding sequence. The line without color referred to introns.

Conserved domains and motifs were supposed to reflect the conservative of *NRT* members. As shown in Figure 3A, the motifs were arranged in a stable order (motif 6, motif 5, motif 1, motif 9, motif 10, motif 2, motif 3, motif 8, motif 4, motif 7) on the NRT protein sequences. Interestingly, some of members, including PtrNPF5.5, PtrNPF5.7, PtrNPF5.8, PtrNPF5.13, PtrNPF5.14, PtrNPF5.15, PtrNPF5.16, and PtrNPF5.19 were not exactly followed with the order. It is probably because the *NPF5* sub-family were experienced several times expansion events. In addition, motif 7 and motif 10 were presented in both NPF and some NRT2 members, implying that these two motifs are potentially connected with the basic function of nitrate or other substrates’ transportation. In addition, *NPF* sub-family had varied superfamily domain regions, including MFS-NPF1-2, MFS-NPF3, MFS-NPF4, MFS-NPF5, MFS-NPF, MFS-NPF7, and MFS-NPF8 (Figure 3B). There was a good correspondence between superfamily domains and *NPF* sub-families, which further confirmed the reliability of the phylogenetic tree. Besides, the PLN00028 and *NAR2* conserved domains were specific for *NRT2* and *NAR2*, respectively. The conserved domains were basically consistent with the re-confirmation results from the SMART database assay (Figure S5). However, the NRT2.5 showed a different pattern with more transmembrane domains. More than that, PtrNPF5.8 and PtrNPF5.19 had a unique conserved domain region UPF0114. For gene structure analysis, some of the genes contained the long fragment of introns, like *PtrNPF1.5*, *PtrNPF2.2*, *PtrNPF5.2*, and *PtrNPF5.3* (Figure 3C). Those long non-coding fragments might have potential roles in the regulation of gene expression. Furthermore, approximately two-thirds of genes had both up and downstream UTRs, indicating that *NRTs* probably had diversified regulation mechanisms. To further detect the regulation factors and predict the *cis*-acting elements of *PtrNRTs*, 2000 bp promoter sequences upstream of the gene start codon were extracted from the genome file according to the gene annotations (Figure S4A). The heat map was constructed to show the frequency of occurrence of different *cis*-acting elements (Figure S4B). According to the result, ABA regulatory *cis*-acting elements and MeJA regulatory *cis*-acting elements were largely distributed in the promoter regions of *PtrNRTs*. Not only that, but there are other hormone-related elements have also been identified, like auxin, salicylic acid, and jasmonic acid. Thus, it is suspected that hormones play potential roles in the regulation of *PtrNRT* genes’ expression. Overall, the *cis*-acting element analysis will provide a good reference for further transcriptional regulation research on *PtrNRT* members.

### 3.4. Expression Analysis of the PtrNRT Genes in Different Tissues

**Figure 4:**
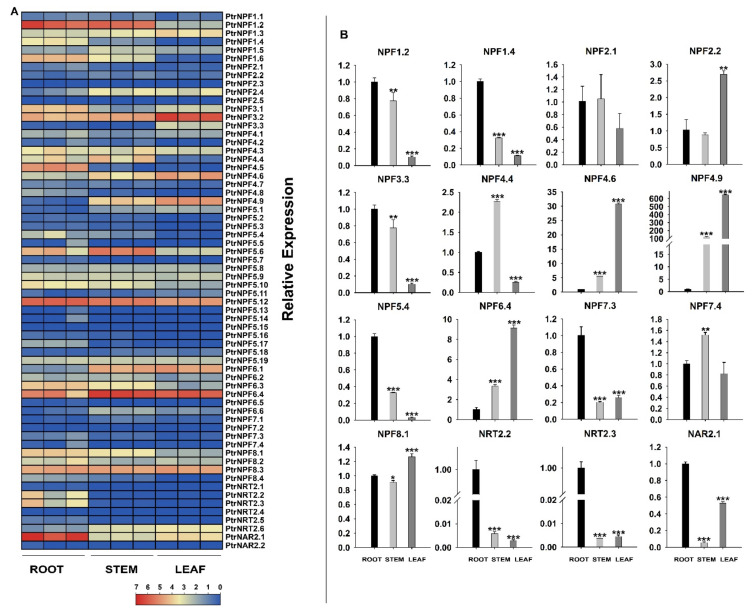
Differential gene expression of *PtrNRTs* in a variety of tissue. (**A**) The FPKM value of 64 *PtrNRT* genes in a heatmap. (**B**) The relative expression of selected *PtrNRT* members by qRT-PCR assay. The root tissue was defined to be the reference. * Represented significant differences in comparison with root group using Student’s *t*-test at 0.01 < *p* < 0.05. ** Represented significant differences at 0.001 < *p* < 0.01. *** Represented significant differences at *p* < 0.001.

The spatio-temporal expression is a key aspect that determines gene function. The expression pattern of *NRT* genes in differential tissues was detected via transcriptome data and a qRT-PCR experiment (Figure 4, Table S2). The RNA-seq result suggested that *NRT* genes were widely expressed in leaf, stem, and root tissues (Figure 4A, Figure S2). Several genes, like *PtrNPF1.2*, *PtrNPF3.3*, *PtrNPF4.9*, and *PtrNPF6.4*, were expressed actively in two or three different tissues. Those genes probably performed basic N element transport functions in the organism. Meanwhile, some of the genes were specifically expressed at one certain tissue, speculating that those genes likely had the given function of loading and discharging nitrate or other substrates. However, some members were not detected, such as *PtrNPF2.1, PtrNPF2.2, PtrNPF5.4,* and *PtrNPF7.4*. It implied that those genes might be expressed at a specific developmental stage or respond to certain stimulations. To further verify the tissue expression results, some *NRTs* were selected for qRT-PCR assay (Figure 4B). The expression of most selected genes was consistent with the transcriptome results. For *PtrNPF6.4*, it was highly expressed in leaf and stem tissues, suggesting that *PtrNPF6.4* should participate in the long-distance transportation of nitrate from root to over-ground part. Meanwhile, *PtrNPF5.4* and *PtrNPF7.3* were highly expressed in root, implying their potential roles in nitrate uptake from soil. The expression pattern in different tissues demonstrated various functions of *PtrNRTs* in the whole plant.

### 3.5. GO and KEGG Pathway Enrichment Analysis under Salt Treatment in P. trifoliata

**Figure 5:**
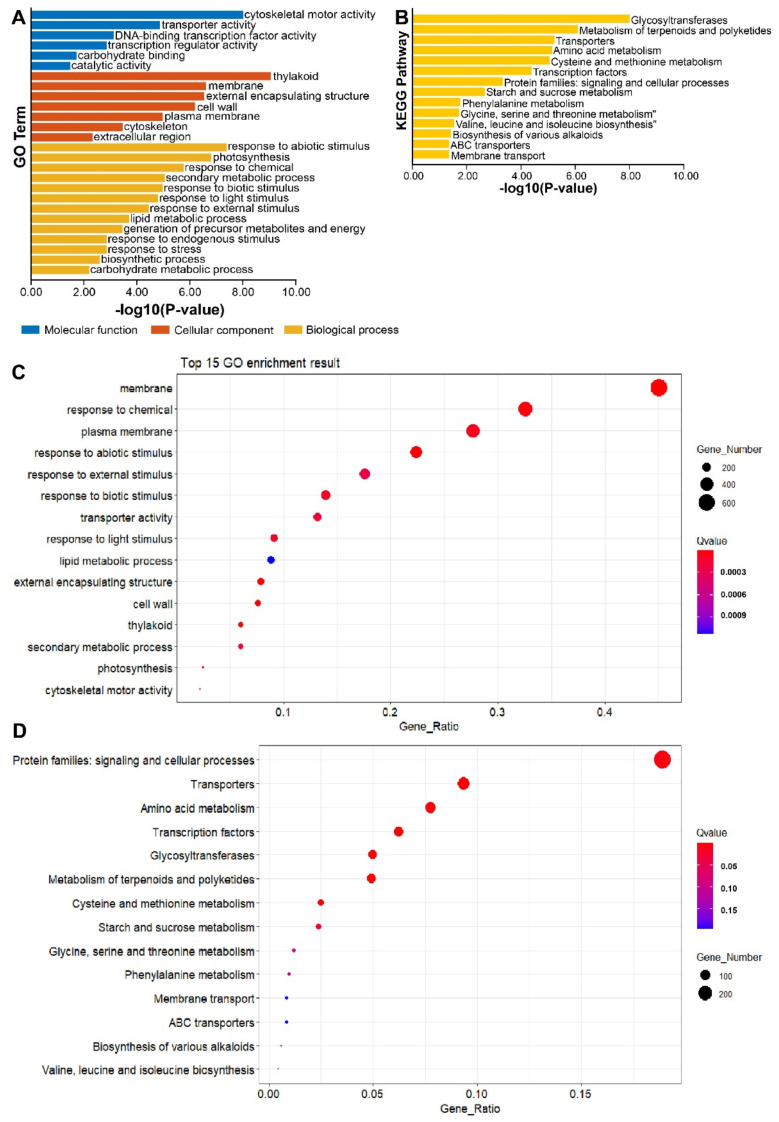
GO and KEGG pathway enrichment plot. The GO (**A**) and KEGG (**B**) enrichment plots were constructed by TBtools. Both GO and KEGG enrichment *p*-value of pathways were verified by the BH method. In addition, the bubble plots used the bubble shiny plugin (**C**,**D**). The circle color represented the enrichment degree, and the size of the circle represented the differential genes participating in a certain pathway.

To get insight into the response of *NRTs* under salt stress, differential expression genes (DEGs) were analyzed from transcriptome data. The GO and KEGG annotation were performed by the eggNOG-mapper. The visualization of enrichment results was carried out containing the bar plots (Figure 5A, B) and the bubble plots (Figure 5C, D). Based on the previous report, transporters play an important role in salt tolerance [48]. Accordingly, we found that some transporter gene clusters were enriched from the molecular function part in GO enrichment results (Figure 5A). The KEGG enrichment result revealed that some amino acid metabolism pathways were enriched, like cysteine, methionine, glycine, serine, threonine, valine, leucine, and isoleucine (Figure 5B). The accumulation of amino acids should work as an osmotic adjustment substance to release salt stress. It is well known that NRT is the transporter of nitrogen compounds and some anions. These results suggested that, at least from the perspective of nitrogen metabolites, NRT is extremely like to be involved in the regulation of salt stress response. In addition, salt stress can also be perceived by plants as abiotic stress signals, resulting in subsequent transcriptional regulation. It inspired us to identify further the expression changes of *PtrNRT* members under salt conditions.

### 3.6. Expression Analysis of the PtrNRT Genes in Response to Salt Stress

**Figure 6:**
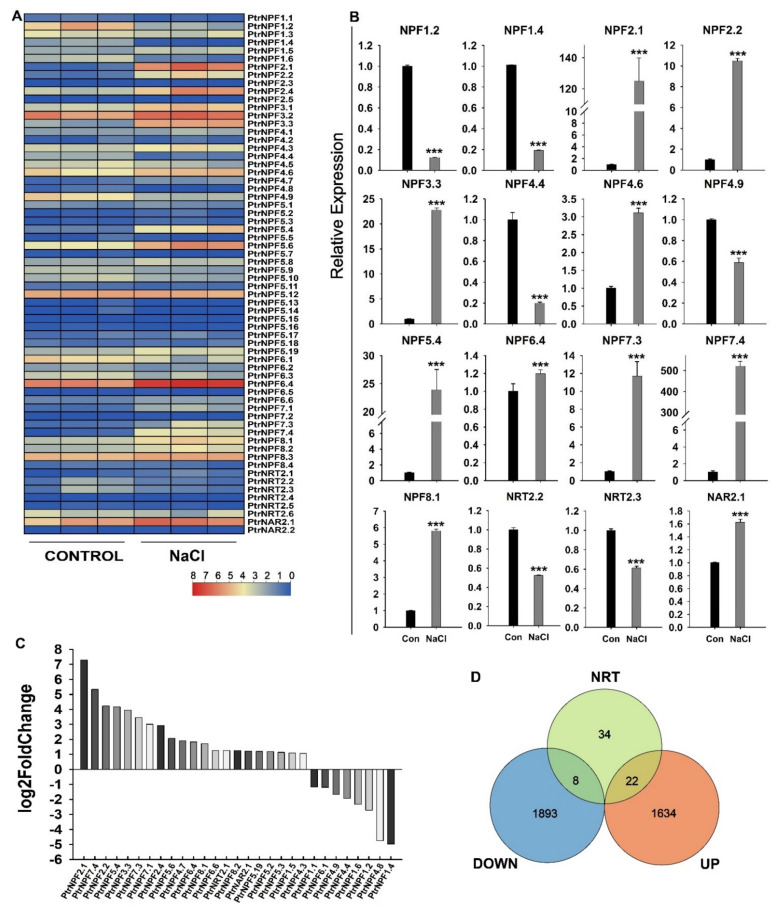
Differential gene expression of *PtrNRTs* under salt conditions. (**A**) The FPKM value from the control and NaCl treatment group. Each group had three biological repetitions. Heat map data was selected from RNA-seq (Table S3,Table S4). (**B**) The selected genes’ expression by qRT-PCR assay. The relative expression was calculated by 2^−∆∆CT^ method. *** Represented significant difference in comparison with control group using Student’s *t*-test at *p* < 0.001. (**C**) The representative differential expression genes under salt stress. (**D**) The Venn plot includes the up and down-regulated genes. The green circle represented all the 64 NRT genes. The red circle and the blue circle respectively represented the genes that up and down-regulated under salt stress (log2 Fold Change absolute value > 1).

In general, the specific function of a gene is closely related to its expression level. The salt-treated transcriptome data were applied to analyze the response of *PtrNRTs* under salt stress. As shown in Figure 6A,C, the expression of *PtrNRTs* had a huge changing tendency in response to salt conditions. The transcriptome results suggested that approximately half of the *NRT* members were induced or inhibited after salt treatment. A total of 22 *NRT* genes were dramatically up-regulated and 8 *NRTs* were down-regulated according to the log2 Fold Change values (Figure 6D). Among them, *PtrNPF6.4*, *PtrNPF2.1*, *PtrNPF3.3*, *PtrNPF7.3*, and *PtrNPF7.4* were obviously induced by the salt treatment. To further confirm the transcriptome result, representative genes were selected for qRT-PCR assay, including the down-regulated genes *PtrNPF1.2, PtrNPF1.4, PtrNPF1.6, PtrNPF4.8* and the up-regulated genes *PtrNAR2.1, PtrNPF6.4, PtrNPF7.3, PtrNPF7.4, PtrNPF2.1, PtrNPF2.2, PtrNPF3.3,* and *PtrNPF5.4* (Figure 6B). Consistently, *PtrNPF2.1*, *PtrNPF5.4*, and *PtrNPF7.4* were up-regulated to a huge degree. In addition, the supposed down-regulate genes like *PtrNPF1.2*, *PtrNPF1.4*, and *PtrNPF4.8* were inhibited according to the qRT-PCR assay (Figure 6B). Taken together, the dramatic changes in *NRT* members’ expression under salt stress implied the potential relationship between nitrogen transportation and salt stress response.

## 4. Discussion

Nitrogen metabolism is one of the basic biological processes in plants, which involves a variety of enzymes and transporters. Among transmembrane carriers, NRTs are mainly involved in nitrate absorption and transportation in plants. In general, *NRT* members are divided into three families, *NPF*, *NRT2*, and *NAR2*. A total of 61 *NRT* genes (53 *NPFs*, 7 *NRT2s*, and 1 *NAR2*) had been identified in *A. thaliana*, 67 *NRT* genes (57 *NPFs*, 9 *NRT2s*, and 1 *NAR2*) were identified in spinach [11], 79 *NRT* genes (68 *NPFs*, 6 *NRT2s*, and 5 *NAR2s*) were identified in poplar [12], 84 *NRT* genes (73 *NPFs*, 5 *NRT2s*, and 2 *NAR2s*) were identified in apple [13]. Here, 64 *NRT*s were explored in the whole genome of *P. trifoliata* (Table 1), including 56 *NPFs*, 6 *NRT2s*, and 2 *NAR2s*. The number of *NRTs* in *P. trifoliata* was similar to other species, implying the relative stability of *NRT* family members throughout the evolutionary process. Moreover, the conserved domain and motif analysis were carried out to reveal further the relationship between the various members and the potential functions (Figure 3). One interesting point is that PtrNRT2.2 and PtrNRT2.3 share almost the same protein sequence. For the coding region, there are only a few nucleotide variations between two members. Moreover, both *NRT2.2* and *NRT2.3* are located at chromosome 3 (Figure 2) and possess almost the same expression pattern (Figure 4 and Figure 6), suggesting that they probably underwent the gene duplication event. The various motifs between *NPF* and *NRT2* genes indicate the variety of molecular functions between high-affinity and low-affinity transport systems. In particular, motif 7 and motif 10 are presented in almost all NPF and NRT2 members, suggesting the essential role of these two domains in nitrate transportation. Motif 7 and motif 10 are respectively located at the C-terminus and the middle of NPF members, while both motif 7 and motif 10 have existed at the end of all NRT2 family members. In addition, the upstream 2000 nucleotides promoter sequences of *PtrNRTs* were extracted to perform *cis*-acting element analysis (Figure S4). The predicted results suggested that *NRTs* might be induced by plant hormones, like methyl jasmonate and abscisic acid. Some *NRT* promoters (including *NPF7.3*, *NPF7.4*, *NPF8.2*, and *NPF8.3*) were also found to possess the MYBHv1 binding sites, suggesting that those genes possibly share the same transcript regulation mechanism. 

The function and regulation mechanism of *NRTs* in nitrate transportation were extensively reported. For example, 16 out of the 53 *NPF* genes have been performed functional verification in *A. thaliana*, which are classified into nitrate transporters and dipeptide metabolite transporters [16,17,49,50,51,52,53,54,55,56,57,58,59]. The *NPFs* belong to a low-affinity transport system except for *AtNRT1.1*, which possesses dual affinity [60]. NRT2s, the high-affinity transporters, have been reported to combine with membrane-targeted NAR2 proteins to enhance transport activity [15,61,62,63,64]. According to the expression pattern of *PtrNRTs* in different tissues (Figure 4), it could be found that *PtrNRTs* are widely expressed in whole plants, including root, stem, and leaf tissues. Whereas some genes are preferentially expressed in a specific tissue, indicating that they might be responsible for special transport functions. In addition, we performed the qRT-PCR assay for all *NRT2* and *NAR* genes under the low nitrogen condition. The result suggested that the high-affinity transport system members were significantly induced by low nitrogen conditions in the root (Figure S3). In general, it could be concluded that *PtrNRTs* have potential functions in nitrogen uptake and transportation in *P. trifoliata* but need more verification in further work. 

A growing body of reports suggested that the absorption, transportation, and assimilation of nitrogen were involved in plants’ salt resistance [34,35,38,40]. Accordingly, the expression of *PtrNRTs* were detected under salt condition. The results demonstrated that 30 out of 64 *PtrNRT* genes’ expression had significant alteration under salt treatment based on the RNA-seq and qRT-PCR assays (Figure 6). Some *NRT* members, like *PtrNPF2.1*, *PtrNPF2.2*, *PtrNPF7.3*, and *PtrNPF7.4* were expressed at a low level in the control group, but their transcript levels were significantly increased after salt treatment. In addition, some of the repressed *PtrNRT* genes were also detected in response to salt stress, including *PtrNPF1.2*, *PtrNPF1.4*, *PtrNPF4.4*, *PtrNPF4.9*, *PtrNRT2.2*, and *PtrNRT2.3*. The fluctuations of *PtrNRTs*’ expression suspected that they were regulated by specific signals in response to salt stress. Besides, the expression profiles also provided some new sights for the functional research of NRTs. For example, the salt-induced expression of *PtrNAR2.1* suggested that the activity of the high-affinity transport system should be increased under salt stress. However, the *NRT2*, the interaction partner of NAR2, was not up-regulated, implying that PtrNAR2s may have unidentified potential functions in salt response. Given that the specific functions of genes are often related to the expression level [65], we hypothesized that there are two possible ways in which *NRT* is in response to salt tolerance in plants. One is to increase the outflow of salt ions or reduce their absorption. Previous studies suggested that chloride (Cl^−^) and nitrate had substitution transportation mechanisms, and NRTs also had been reported to have chloride ion transport activity [37]. Accordingly, we speculated that the plant might harbor a Cl^−^/NO_3_^−^ cooperative transport mechanism via the regulation of *NRTs*, which is important for salt resistance, especially for the Cl^−^ sensitive plant. The other pathway is to improve nitrogen metabolism, which finally accumulates more osmotic regulatory substances like amino acids to alleviate salt stress. Consistently, many amino acid metabolism pathways were clustered under salt conditions (Figure 5), which were potentially related to the up-regulated expression of *NRTs* for higher efficient nitrogen metabolism under salt stress. In conclusion, this study identified the *NRT* genes in P. trifoliata and detected their expression patterns under salt stress and in different tissues, which provided a significant reference for further functional research of PtrNRTs to improve the plant nitrogen use efficiency under salt stress.

## Figures and Tables

**Table 1:** Characteristics of PtrNRT members.

Accession Number ^1^	Gene ID	No. of AA ^2^	Start Site ^3^	End Site ^3^	MW (kDa) ^4^	pI ^5^	GRAVY ^6^
*Pt1g007360*	*PtrNPF1.1*	585	6701392	6706813	64.94	9.24	0.316
*Pt7g010530*	*PtrNPF1.2*	625	12932806	12936421	68.80	8.47	0.293
*Pt1g005100*	*PtrNPF1.3*	572	3120786	3128240	62.63	8.73	0.282
*Pt1g007370*	*PtrNPF1.4*	594	6707620	6709906	65.68	8.73	0.200
*Pt8g002750*	*PtrNPF1.5*	576	1842390	1858910	63.44	8.50	0.311
*Pt1g005080*	*PtrNPF1.6*	581	3134219	3137466	63.91	7.44	0.265
*Pt3g001010*	*PtrNPF2.1*	588	1262243	1264918	65.37	9.36	0.224
*Pt3g001000*	*PtrNPF2.2*	621	1247077	1258790	68.69	9.03	0.188
*Pt3g001020*	*PtrNPF2.3*	599	1271264	1273237	66.08	9.05	0.249
*Pt4g003350*	*PtrNPF2.4*	615	2043405	2047306	68.49	9.18	0.224
*Pt6g014330*	*PtrNPF2.5*	589	10127132	10129206	65.21	9.09	0.225
*Pt6g013450*	*PtrNPF3.1*	874	9410246	9415048	96.73	6.48	0.199
*Pt4g008540*	*PtrNPF3.2*	581	6165780	6169493	64.65	8.98	0.250
*Pt4g008550*	*PtrNPF3.3*	581	6155010	6158555	64.31	8.90	0.285
*Pt2g029890*	*PtrNPF4.1*	895	28694854	28703982	98.89	7.25	0.204
*Pt4g012130*	*PtrNPF4.2*	524	9062853	9065920	58.15	9.05	0.328
*PtUn015880*	*PtrNPF4.3*	605	31889734	31893237	67.43	9.15	0.140
*Pt3g020700*	*PtrNPF4.4*	614	25583942	25587711	68.26	8.94	0.283
*Pt5g003870*	*PtrNPF4.5*	588	2587369	2590412	65.06	8.70	0.329
*Pt4g003240*	*PtrNPF4.6*	579	1982178	1987204	63.48	8.17	0.428
*Pt3g001060*	*PtrNPF4.7*	577	1312638	1315969	63.72	9.13	0.383
*PtUn010670*	*PtrNPF4.8*	616	21086318	21089327	67.70	7.03	0.256
*PtUn010660*	*PtrNPF4.9*	597	21037830	21048570	65.82	8.14	0.263
*Pt4g020770*	*PtrNPF5.1*	584	20356772	20360906	65.32	9.16	0.200
*Pt9g008600*	*PtrNPF5.2*	633	6405423	6415253	70.97	9.13	0.262
*PtUn035060*	*PtrNPF5.3*	595	67666483	67683281	66.58	9.18	0.237
*PtUn007180*	*PtrNPF5.4*	610	11116991	11120542	67.74	9.00	0.326
*Pt6g013470*	*PtrNPF5.5*	488	9424535	9426524	55.00	8.26	0.433
*Pt6g014250*	*PtrNPF5.6*	592	10060119	10064466	65.62	8.88	0.332
*Pt6g013460*	*PtrNPF5.7*	570	9416116	9423292	63.61	5.56	0.430
*Pt5g016390*	*PtrNPF5.8*	777	12824326	12831040	86.71	9.18	0.295
*Pt2g008110*	*PtrNPF5.9*	535	4826115	4830066	59.83	8.75	0.354
*Pt5g005040*	*PtrNPF5.10*	558	3350358	3352696	61.58	8.01	0.412
*PtUn026920*	*PtrNPF5.11*	498	54785836	54789355	55.57	9.61	0.225
*Pt5g005050*	*PtrNPF5.12*	569	3357900	3361719	62.83	6.95	0.369
*Pt9g009730*	*PtrNPF5.13*	431	7757020	7758583	48.69	8.30	0.132
*Pt9g009670*	*PtrNPF5.14*	448	7703996	7706735	50.57	8.80	0.186
*PtUn005790*	*PtrNPF5.15*	567	7939272	7942114	64.07	9.13	0.451
*Pt9g009790*	*PtrNPF5.16*	788	7812307	7815445	87.66	8.56	0.231
*Pt9g008580*	*PtrNPF5.17*	595	6395222	6398328	66.54	9.11	0.208
*Pt3g030260*	*PtrNPF5.18*	606	35941239	35944759	67.20	9.20	0.243
*PtUn028140*	*PtrNPF5.19*	628	56454824	56461168	70.25	9.42	0.370
*PtUn008620*	*PtrNPF6.1*	634	14160194	14162884	70.31	8.83	0.238
*Pt1g006350*	*PtrNPF6.2*	581	2152543	2155882	63.49	9.08	0.339
*Pt3g008120*	*PtrNPF6.3*	553	6492850	6496785	60.95	9.10	0.263
*Pt2g013780*	*PtrNPF6.4*	588	17449466	17451952	65.33	9.13	0.226
*Pt5g002490*	*PtrNPF6.5*	515	1693500	1693532	56.93	5.60	0.239
*Pt1g003070*	*PtrNPF6.6*	579	4853921	4857900	63.50	9.20	0.409
*Pt2g001610*	*PtrNPF7.1*	504	1224993	1228021	55.40	9.00	0.339
*Pt3g039860*	*PtrNPF7.2*	602	42571348	42574088	66.47	7.90	0.199
*Pt4g015230*	*PtrNPF7.3*	596	13417384	13421889	66.00	6.39	0.265
*Pt3g041260*	*PtrNPF7.4*	613	43574897	43579506	68.08	8.40	0.213
*Pt6g013550*	*PtrNPF8.1*	568	9499282	9503587	63.40	8.82	0.214
*Pt8g011520*	*PtrNPF8.2*	574	9266295	9270180	64.15	6.76	0.182
*Pt2g017400*	*PtrNPF8.3*	584	20631552	20636512	64.40	5.96	0.303
*Pt6g013540*	*PtrNPF8.4*	501	9494530	9496538	55.98	8.58	0.246
*Pt8g007700*	*PtrNRT2.1*	535	5808930	5811379	57.99	9.14	0.343
*Pt8g007710*	*PtrNRT2.2*	530	3120817	3122747	57.43	9.35	0.374
*Pt8g007720*	*PtrNRT2.3*	530	6554577	6556169	57.39	9.35	0.382
*Pt6g017660*	*PtrNRT2.4*	530	6537447	6539366	57.48	9.22	0.392
*Pt4g006700*	*PtrNRT2.5*	419	6559711	6561547	45.27	9.18	0.392
*Pt2g007200*	*PtrNRT2.6*	471	15634625	15636904	50.91	8.96	0.456
*Pt3g004750*	*PtrNAR2.1*	173	4426662	4427403	19.23	9.37	−0.479
*Pt4g006240*	*PtrNAR2.2*	170	3424356	3425077	19.14	6.83	0.002

^1^ Accession number consisted of the CPBD. ^2^ Number of AA represented the number of the amino acids in each protein sequence. ^3^ Start site and end site were found in the gene structure file download in CPBD. ^4^ MW (kDa) was the molecular weight. ^5^ The pI was the theoretical isoelectric point. ^6^ GRAVY was the Grand average of hydropathicity. The number of amino acids, molecular weight, theoretical isoelectric point, and the grand average of hydropathicity were calculated and predicted on the Expasy website (https://web.expasy.org/protparam/, accessed on 1 March 2022).

## Data Availability

The *A. thaliana* and *P. trifoliata* protein sequences were downloaded from the *A. thaliana* information source (TAIR) database (https://www.arabidopsis.org/, accessed on 2 March 2022) and Citrus Pan-genome to Breeding Database (CPBD, http://citrus.hzau.edu.cn/index.php, accessed on 1 March 2022).

## Supplementary Materials

The following supporting information can be downloaded at: https://www.mdpi.com/article/10.3390/genes13071115/s1. Supplementary Materials contained Table S1A about the dosage of various ingredients of hydroponic culture solution, Table S1B is the low nitrogen treatment formula. Table S2 was about the primer pairs for the qRT-PCR experiment. Tables S3 and S4 contained the FPKM value of *NRT* genes from transcriptome data. Figure S1 shows detailed information about the motifs of *the NRT* gene. Figure S2 was the FPKM value-stack figure, which suggested the tissue differential expression. Figure S3 presented the expression of *NRT2* and *NAR* genes under the low nitrogen condition. Figure S4 is the *cis*-acting elements of *PtrNRT* genes. Figure S5: Smart domain visualization. Text S1 was the protein sequence of 64 PtrNRT members. Text S2 was the upstream promoter sequence of 64 *PtrNRT* genes.
