# Supplementary material for: Genome-Wide Identification of NRT Gene Family and Expression Analysis of Nitrate Transporters in Response to Salt Stress in Poncirus trifoliata
Source: Genes (Basel). 2022 Jun 22;13(7):1115. doi: 10.3390/genes13071115 (PMC9323722; doi:10.3390/genes13071115)
Supplement: Supplementary file 1 [file genes-13-01115-s001.zip › supplementary materials .pdf]

Table S1. Nutrition formula for plant cultivation.

**A. Normal formula**	
KNO_3	2 mM
Ca $(NO_3)_2$	1.5 mM
$MgSO_4$	0.5 mM
Na2HPO$_4$	0.14 mM
NaH_2PO_4	0.32 mM
$MnCl_2$	9.15 µM
$ZnSO_4$	1.6 µM
$CuSO_4$	0.32 µM
Na_2MoO_4	0.36 µM
Fe-EDTA	37.3 µM
H_3BO_3	10µM
**B. Low nitrogen formula**	
KNO_3	0.1 mM
K_2SO_4	0.95 mM
$CaCl_2$	1.5 mM
$MgSO_4$	0.5 mM
Na2HPO$_4$	0.14 mM
NaH_2PO_4	0.32 mM
$MnCl_2$	9.15 µM
$ZnSO_4$	1.6 µM
$CuSO_4$	0.32 µM
Na_2MoO_4	0.36 µM
Fe-EDTA	37.3 µM
H_3BO_3	10µM

Table S2. Primers for qRT-PCR assay.

PtrNPF1.2-qPCR-F	CCACAGCATTGCTTGAACGG
PtrNPF1.2-qPCR-R	CTAGACCAAACAGGGAGGCA
PtrNFP1.4-qPCR-F	GATTGGTTTGGCCGAGGCTT
PtrNFP1.4-qPCR-R	TGCTAGACATGCTCTTGGGA
PtrNFP2.1-qPCR-F	TCTTCAGAGAATGGCCATCGG
PtrNFP2.1-qPCR-R	TAAACCCACAGGCCTGGAAAG
PtrNFP2.2-qPCR-F	CCCATTTGTCCGAAGGCTCA
PtrNFP2.2-qPCR-R	ACGGTAGGCTTGGTAAGAGC
PtrNFP3.3-qPCR-F	AGTCTTCACGGGATAGCCGA
PtrNFP3.3-qPCR-R	CCCAATCGAATTCGCAGTCC
PtrNPF4.4-qPCR-F	CCCCGTTGCATCGAAATTCAC
PtrNPF4.4-qPCR-R	CTATTAGGGCAGCTGCAACC
PtrNPF4.6-qPCR-F	TTCTTCACAGAGGCACCGAC

PtrNPF4.6-qPCR-R	TGGTTTGTTCCCCGAATGGT
PtrNPF4.9-qPCR-F	CGGCATGAAAACGCTTGCT
PtrNPF4.9-qPCR-R	CAACCATCCTTGTTTGCTGGG
PtrNFP5.4-qPCR-F	AGTAGAAGCCAAAAGGGTCCG
PtrNFP5.4-qPCR-R	TGGGAGTAGCCACCAAATGC
PtrNPF6.4-qPCR-F	CTGTGGAGCAGGCAACCTTC
PtrNPF6.4-qPCR-R	TGATTTTTCGAGCCAGGGGAA
PtrNFP7.3-qPCR-F	AACATTGTGAAGGCGCAAGC
PtrNFP7.3-qPCR-R	GCTCTTTAAGCCATCTGGTGC
PtrNFP7.4-qPCR-F	CAACCTTCCACATCCCACCG
PtrNFP7.4-qPCR-R	GTGGGTCTAGAACTCGCCTG
PtrNPF8.1-qPCR-F	CGATTTTCTGGCAAGTACCGC
PtrNPF8.1-qPCR-R	TGATAGAGCCGAGCACAAGC
PtrNRT2.1-qPCR-F	AGGGAACTTCGGTTCGGGAT
PtrNRT2.1-qPCR-R	GAGAGTGCAAGCCACAGTCA
PtrNRT2.2-qPCR-F	TGCATCAAGGAAGTCTCCGAT
PtrNRT2.2-qPCR-R	TGGAAGGTGTAGTATTTGGGGGT
PtrNRT2.3-qPCR-F	GTCTCCGATTCGCCGAGAAC
PtrNRT2.3-qPCR-R	GGCAGGTGTAGTATTTGGGGG
PtrNRT2.4-qPCR-F	TTCCAGACACTCGACGGCTA
PtrNRT2.4-qPCR-R	GAAAATGCACCAAGGCCACC
PtrNRT2.5-qPCR-F	ACAGGGATCACTCTCATGGGT
PtrNRT2.5-qPCR-R	CAGAACATTCCGCCCCATTG
PtrNRT2.6-qPCR-F	TGTGTGTGTTGCTTGGACGA
PtrNRT2.6-qPCR-R	CGGCAGCTTGAACGAACAC
PtrNAR2.1-qPCR-F	CTCTTAACGCCGAACGTCAC
PtrNAR2.1-qPCR-R	GACGCTGAAGCAAACGGATG
PtrNAR2.2-qPCR-F	TGCAATTAATGGGCGGCATC
PtrNAR2.2-qPCR-R	ATCCAAACAAAGACACCGCAG
ACTIN- qPCR-F	CCGACCGTATGAGCAAGGAAA
ACTIN- qPCR-R	TTCCTGTGGACAATGGATGGA

Table S3. FPKM value of PtrNRT genes from RNA-seq data in different tissues.

Tissue	Name	con_root1	con_root2	con_root3	con_stem1	con_stem2	con_stem3	con_leaf1	con_leaf2	con_leaf3
Pt1g007360.1	*PtrNPF1.1*	0.82	1.04	1.44	1.28	0.77	1.33	0.22	0.8	0.59
Pt7g010530.1	*PtrNPF1.2*	90.27	61.16	46.65	57.87	50.75	44.89	4	3.4	3.99
Pt1g005100.1	*PtrNPF1.3*	5.66	6.64	5.71	7.5	8.5	9.21	14.38	10.16	11.63
Pt1g007370.1	*PtrNPF1.4*	9.74	11	8.11	1.48	1.85	1.41	0.05	0.09	0.24
Pt8g002750.4	*PtrNPF1.5*	2.61	2.97	1.82	6.89	4.59	5.22	2.84	2.66	3.13
Pt1g005080.1	*PtrNPF1.6*	19.37	18.6	18.69	9.46	6.95	6.72	0.25	0.23	0.14
Pt3g001010.1	*PtrNPF2.1*	0.64	1.03	1.77	0.5	0.3	0.19	0.19	0.43	0.24
Pt3g001000.1	*PtrNPF2.2*	0.6	0.35	0.71	1.31	0.72	1.48	0.68	0.43	0.69
Pt3g001020.1	*PtrNPF2.3*	0	0	0.03	0	0	0	0	0	0
Pt4g003350.1	*PtrNPF2.4*	0.78	1.57	3.06	9.49	6.44	9.37	7.9	5.62	9.21
Pt6g014330.1	*PtrNPF2.5*	0	0	0	0	0	0	0	0	0
Pt6g005490.1	*PtrNPF3.1*	16.82	14.83	17.66	3.37	1.37	2.2	5.63	5.99	5.5
Pt4g008540.1	*PtrNPF3.2*	23.91	16.65	19.01	23.08	23.98	26.71	89.53	64.16	68.07
Pt4g008550.1	*PtrNPF3.3*	0.25	0.23	0.23	0	0.1	0.15	6.47	4.88	5.52
Pt2g029890.1	*PtrNPF4.1*	3.37	2.68	4.27	1.61	1.31	1.82	2.94	2.82	2.72
Pt4g012130.1	*PtrNPF4.2*	0.44	0.34	1.78	0.49	0.35	0.84	0	0.05	0.03
PtUn015880.1	*PtrNPF4.3*	9.81	13.79	7.19	7.56	5.45	12.38	6.79	5.76	5.88
Pt3g020700.1	*PtrNPF4.4*	7.04	14.02	4.85	19.39	7.64	17.91	0.48	0.64	0.57
Pt5g003870.1	*PtrNPF4.5*	28.78	35.09	28.41	0.46	0.64	0.03	0.16	0.18	0
Pt4g003240.1	*PtrNPF4.6*	5.61	5.02	3.53	14.58	9.4	14.26	27.7	20.63	29.16
Pt3g001060.2	*PtrNPF4.7*	1.23	1.28	0.81	1.21	0.42	0.84	0.49	0.28	0.42
PtUn010670.1	*PtrNPF4.8*	3.17	2	1.67	0.17	0.45	0.17	0.03	0	0
PtUn010660.3	*PtrNPF4.9*	0.5	0.26	0.13	12.8	16.09	13.35	30.19	31.39	28.16
Pt4g020770.1	*PtrNPF5.1*	0.38	0.32	0.11	1.84	2.32	2.19	3.67	3.16	2.56
Pt9g008600.1	*PtrNPF5.2*	0.35	0.53	0.43	0.29	0.4	0.38	0	0.03	0.12
PtUn035060.1	*PtrNPF5.3*	0.51	0.44	0.59	0.24	0.36	0.28	0	0.03	0.08
PtUn007180.1	*PtrNPF5.4*	4.22	7.52	1.97	2.18	1.14	1.5	0.11	0.23	0.04
Pt6g013470.1	*PtrNPF5.5*	0.16	0.07	2.67	0	0	0	0	0	0

Pt6g014250.1	*PtrNPF5.6*	18.97	23.57	7.4	44.67	35.96	44.09	4.97	5.58	6.28
Pt6g013460.1	*PtrNPF5.7*	0.4	0.4	0.57	0	0	0	0	0	0
Pt5g016390.1	*PtrNPF5.8*	3.14	3.16	2.8	4.14	3.71	3.4	3.98	3.83	2.88
Pt2g008110.1	*PtrNPF5.9*	6.63	5.19	4.87	5.86	5.03	5.18	4.06	5.56	5.31
Pt5g005040.1	*PtrNPF5.10*	11.47	9.72	10.08	6.86	8.71	6.2	2.92	3.34	2.67
PtUn026920.1	*PtrNPF5.11*	0.64	0.1	0.2	0.52	0.32	0.64	1.2	0.79	1.37
Pt5g005050.1	*PtrNPF5.12*	54.78	57.49	58.06	43.32	41	41.47	26.74	28.15	27.79
Pt9g009730.1	*PtrNPF5.13*	0	0	0.76	0	0	0	0	0	0
Pt9g009670.1	*PtrNPF5.14*	0	0	1.75	0	0	0	0	0	0
PtUn005790.1	*PtrNPF5.15*	0	0	0	0	0	0	0	0	0
Pt9g009790.1	*PtrNPF5.16*	0.55	0.36	0.45	0.2	0.35	0.1	0	0.04	0.03
Pt9g008580.1	*PtrNPF5.17*	2.92	2.99	2.62	0.07	0.29	0.04	0.05	0.05	0.02
Pt3g030260.1	*PtrNPF5.18*	0.29	0.28	0.22	0.34	0.78	0.74	0.95	0.52	1
PtUn028140.1	*PtrNPF5.19*	4.92	4.67	5.75	5.37	5.45	4.93	5.38	4.48	4.59
PtUn008620.5	*PtrNPF6.1*	1.6	1.47	1.98	24.06	20.26	25.73	31.21	23.07	23.68
Pt1g006350.1	*PtrNPF6.2*	4.07	2.49	3.44	3.32	4.41	3.56	1.44	2.63	2.16
Pt3g008120.1	*PtrNPF6.3*	14.94	17.1	15.73	10.73	10.3	10.42	3.54	1.9	3.16
Pt2g013780.1	*PtrNPF6.4*	36.35	41.96	14.69	92.97	86.91	72.83	57.62	67.87	56.55
Pt5g002490.1	*PtrNPF6.5*	0.13	0.1	0.27	0	0	0	0	0	0
Pt1g003070.1	*PtrNPF6.6*	0.15	0.55	0.67	3.98	2.7	3.18	1.44	1.98	1.19
Pt2g001610.1	*PtrNPF7.1*	0.09	0.26	0.1	0.58	0.95	0.75	0.25	0.28	0.26
Pt3g039860.1	*PtrNPF7.2*	0.06	0	0.03	0.02	0.03	0	0	0	0
Pt4g015230.1	*PtrNPF7.3*	0.84	0.98	1.83	0.32	0.5	0.52	0.07	0.14	0.28
Pt3g041260.1	*PtrNPF7.4*	0.25	0.05	1.76	0	0	0	0.11	0.05	0
Pt6g013550.1	*PtrNPF8.1*	15.09	15.54	13.73	10.76	10.16	9.87	2.93	3.48	2.9
Pt8g011520.2	*PtrNPF8.2*	6.16	5.28	11.57	4.36	2.91	2.74	5.06	3.61	3.99
Pt2g017400.1	*PtrNPF8.3*	21.84	25.63	27.87	26.12	25.01	23.15	29.45	24.43	24.25
Pt6g013540.1	*PtrNPF8.4*	2.9	2.33	1.76	0.98	0.49	0.67	0.03	0	0
Pt8g007700.1	*PtrNRT2.1*	0.21	0.15	0.25	0	0	0	0	0	0

Pt8g007710.1	*PtrNRT2.2*	15.3	3.56	9.01	0.1	0.07	0.06	0	0.08	0
Pt8g007720.1	*PtrNRT2.3*	18.21	4.79	10.53	0.19	0.06	0.11	0	0.04	0
Pt6g017660.1	*PtrNRT2.4*	0.02	0	0	0	0	0	0	0	0
Pt4g006700.1	*PtrNRT2.5*	0.9	0.63	0.96	0.09	0.11	0.09	0.01	0	0
Pt2g007200.1	*PtrNRT2.6*	1.34	2.38	1.88	6.89	7.45	7.49	10.07	8.88	9.59
Pt3g004750.1	*PtrNAR2.1*	110.84	65.62	98.66	6.31	7.27	6.14	12.53	12.61	11.29
Pt4g006240.1	*PtrNAR2.2*	0.25	0	0.06	0.07	0	0	0	0	0

Table S4. FPKM value of PtrNRT genes from RNA-seq data in salt treatment.

NaCl treatment	Name	con1	con2	con3	nacl1	nacl2	nacl3
Pt1g007360.1	*PtrNPF1.1*	0.76	1.02	0.74	0.19	0.42	0.18
Pt7g010530.1	*PtrNPF1.2*	19.84	41.28	26.59	3.72	4.65	2.73
Pt1g005100.1	*PtrNPF1.3*	11.91	8.25	9.00	6.90	5.76	10.35
Pt1g007370.1	*PtrNPF1.4*	2.44	3.78	3.37	0.13	0.09	0.03
Pt8g002750.4	*PtrNPF1.5*	3.71	3.26	2.53	6.93	4.89	7.29
Pt1g005080.1	*PtrNPF1.6*	4.48	6.50	7.50	1.08	1.36	0.54
Pt3g001010.1	*PtrNPF2.1*	0.39	0.29	0.81	50.69	101.78	61.44
Pt3g001000.1	*PtrNPF2.2*	0.89	0.54	0.90	9.42	20.89	9.95
Pt3g001020.1	*PtrNPF2.3*	0.00	0.00	0.00	0.06	0.05	0.00
Pt4g003350.1	*PtrNPF2.4*	7.42	4.70	6.08	21.81	61.14	45.73
Pt6g014330.1	*PtrNPF2.5*	0.00	0.00	0.00	0.00	0.00	0.00
Pt6g005490.1	*PtrNPF3.1*	6.84	8.35	7.78	23.60	31.05	21.38
Pt4g008540.1	*PtrNPF3.2*	69.65	38.69	35.56	86.91	105.66	77.47
Pt4g008550.1	*PtrNPF3.3*	4.12	2.16	1.86	37.21	38.37	41.05
Pt2g029890.1	*PtrNPF4.1*	2.70	2.69	2.74	2.74	4.95	3.68
Pt4g012130.1	*PtrNPF4.2*	0.17	0.09	1.12	0.27	0.84	0.33
PtUn015880.1	*PtrNPF4.3*	8.13	6.92	6.81	13.82	18.33	10.44
Pt3g020700.1	*PtrNPF4.4*	3.75	3.85	4.03	0.46	1.10	0.87
Pt5g003870.1	*PtrNPF4.5*	6.55	7.46	11.00	6.26	5.41	2.99
Pt4g003240.1	*PtrNPF4.6*	21.58	13.09	12.91	24.91	27.64	30.14
Pt3g001060.2	*PtrNPF4.7*	0.35	0.39	0.85	2.00	1.65	2.39
PtUn010670.1	*PtrNPF4.8*	0.23	0.72	0.95	0.00	0.00	0.04
PtUn010660.3	*PtrNPF4.9*	22.67	16.46	14.47	4.95	4.40	5.17
Pt4g020770.1	*PtrNPF5.1*	2.72	1.55	1.12	2.33	1.55	1.20
Pt9g008600.1	*PtrNPF5.2*	0.10	0.20	0.18	0.49	0.85	0.15
PtUn035060.1	*PtrNPF5.3*	0.09	0.24	0.19	0.52	0.58	0.26
PtUn007180.1	*PtrNPF5.4*	1.14	1.21	0.86	11.83	15.30	25.95
Pt6g013470.1	*PtrNPF5.5*	0.00	0.03	0.74	0.21	0.03	0.00

Pt6g014250.1	***PtrNPF5.6***	**11.74**	**13.18**	**11.91**	**32.44**	**59.32**	**49.20**
Pt6g013460.1	***PtrNPF5.7***	**0.11**	**0.00**	**0.29**	**0.00**	**0.00**	**0.00**
Pt5g016390.1	***PtrNPF5.8***	**3.27**	**4.07**	**3.48**	**7.67**	**5.32**	**5.98**
Pt2g008110.1	***PtrNPF5.9***	**5.74**	**5.59**	**5.19**	**2.95**	**3.00**	**2.24**
Pt5g005040.1	***PtrNPF5.10***	**4.16**	**6.29**	**7.56**	**4.12**	**3.56**	**2.88**
PtUn026920.1	***PtrNPF5.11***	**0.73**	**0.75**	**0.64**	**0.36**	**0.56**	**0.50**
Pt5g005050.1	***PtrNPF5.12***	**35.40**	**39.81**	**39.89**	**39.48**	**36.35**	**30.59**
Pt9g009730.1	***PtrNPF5.13***	**0.00**	**0.00**	**0.44**	**0.00**	**0.00**	**0.00**
Pt9g009670.1	***PtrNPF5.14***	**0.00**	**0.00**	**0.60**	**0.00**	**0.00**	**0.00**
PtUn005790.1	***PtrNPF5.15***	**0.00**	**0.00**	**0.00**	**0.00**	**0.08**	**0.00**
Pt9g009790.1	***PtrNPF5.16***	**0.08**	**0.18**	**0.17**	**0.03**	**0.00**	**0.06**
Pt9g008580.1	***PtrNPF5.17***	**0.49**	**0.54**	**1.27**	**0.85**	**1.84**	**0.64**
Pt3g030260.1	***PtrNPF5.18***	**0.98**	**0.53**	**0.62**	**0.83**	**0.47**	**1.09**
PtUn028140.1	***PtrNPF5.19***	**4.21**	**4.55**	**5.19**	**11.70**	**8.52**	**9.18**
PtUn008620.5	***PtrNPF6.1***	**22.61**	**16.41**	**16.42**	**8.98**	**2.59**	**7.91**
Pt1g006350.1	***PtrNPF6.2***	**2.53**	**3.47**	**3.09**	**1.67**	**2.29**	**1.90**
Pt3g008120.1	***PtrNPF6.3***	**6.87**	**8.84**	**8.59**	**3.38**	**6.63**	**3.27**
Pt2g013780.1	***PtrNPF6.4***	**61.98**	**64.81**	**44.04**	**168.60**	**208.00**	**177.23**
Pt5g002490.1	***PtrNPF6.5***	**0.00**	**0.00**	**0.03**	**0.11**	**0.00**	**0.00**
Pt1g003070.1	***PtrNPF6.6***	**1.37**	**1.16**	**1.34**	**2.81**	**3.00**	**2.75**
Pt2g001610.1	***PtrNPF7.1***	**0.51**	**0.48**	**0.61**	**3.63**	**5.36**	**3.01**
Pt3g039860.1	***PtrNPF7.2***	**0.00**	**0.00**	**0.03**	**0.07**	**0.00**	**0.04**
Pt4g015230.1	***PtrNPF7.3***	**0.47**	**0.59**	**0.75**	**2.06**	**9.83**	**7.31**
Pt3g041260.1	***PtrNPF7.4***	**0.02**	**0.30**	**0.49**	**11.85**	**11.51**	**8.82**
Pt6g013550.1	***PtrNPF8.1***	**5.62**	**6.43**	**6.96**	**16.68**	**22.31**	**17.76**
Pt8g011520.2	***PtrNPF8.2***	**4.24**	**4.63**	**5.69**	**8.92**	**14.45**	**8.76**
Pt2g017400.1	***PtrNPF8.3***	**29.62**	**26.93**	**26.55**	**37.08**	**36.87**	**36.99**
Pt6g013540.1	***PtrNPF8.4***	**0.50**	**0.90**	**0.91**	**0.84**	**0.72**	**0.24**
Pt8g007700.1	***PtrNRT2.1***	**0.00**	**0.10**	**0.47**	**0.77**	**1.68**	**0.66**
Pt8g007710.1	***PtrNRT2.2***	**0.63**	**4.05**	**2.73**	**1.04**	**1.04**	**0.52**
Pt8g007720.1	***PtrNRT2.3***	**0.62**	**5.27**	**3.39**	**1.47**	**1.16**	**0.64**
Pt6g017660.1	***PtrNRT2.4***	**0.00**	**0.00**	**0.00**	**0.00**	**0.00**	**0.00**
Pt4g006700.1	***PtrNRT2.5***	**0.05**	**0.29**	**0.23**	**0.12**	**0.22**	**0.00**
Pt2g007200.1	***PtrNRT2.6***	**9.51**	**5.69**	**6.38**	**5.18**	**2.54**	**8.75**
Pt3g004750.1	***PtrNAR2.1***	**21.35**	**42.97**	**39.50**	**93.89**	**78.23**	**54.38**
Pt4g006240.1	***PtrNAR2.2***	**0.00**	**0.10**	**0.00**	**0.10**	**0.00**	**0.03**

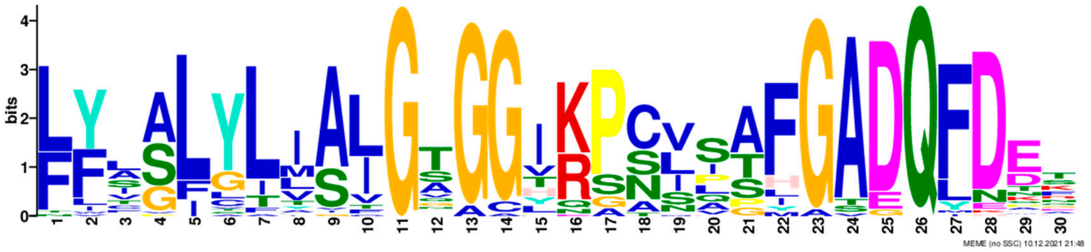

Motif 1

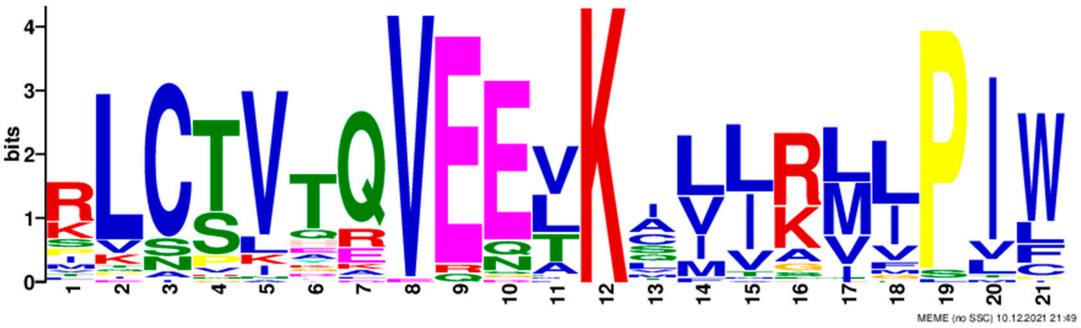

Motif 2

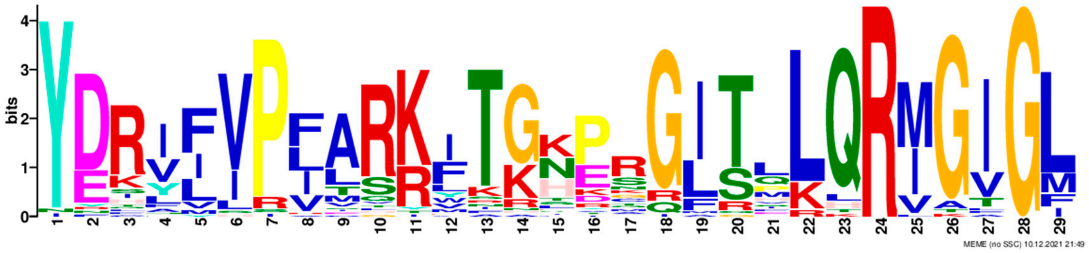

Motif 3

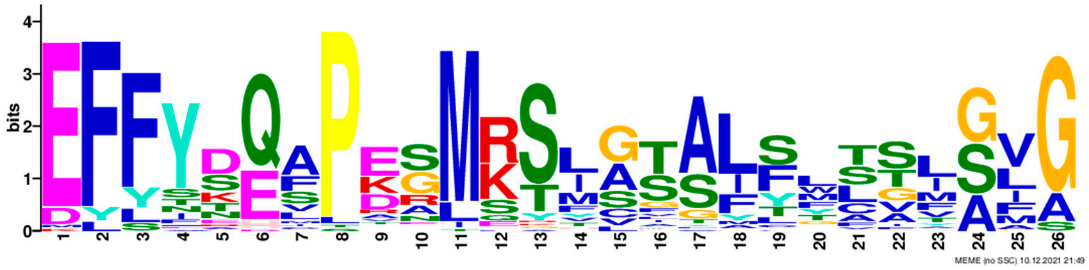

Motif 4

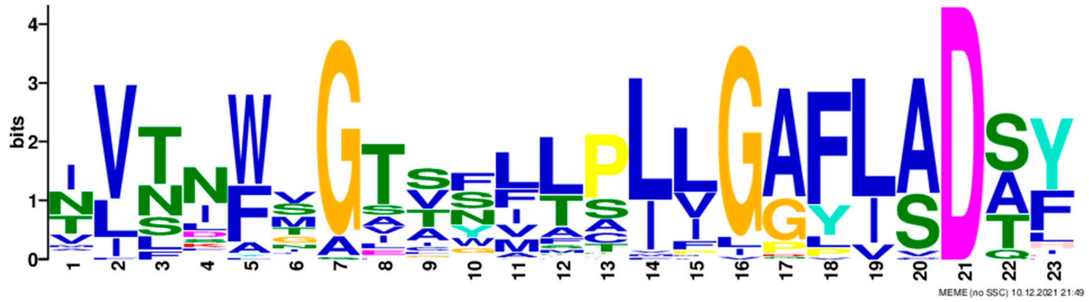

Motif 5

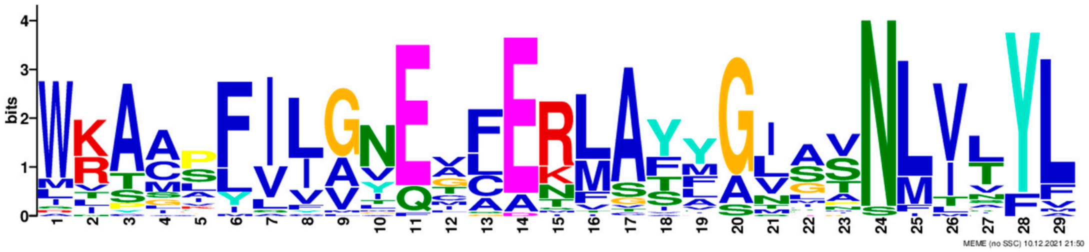

Motif 6

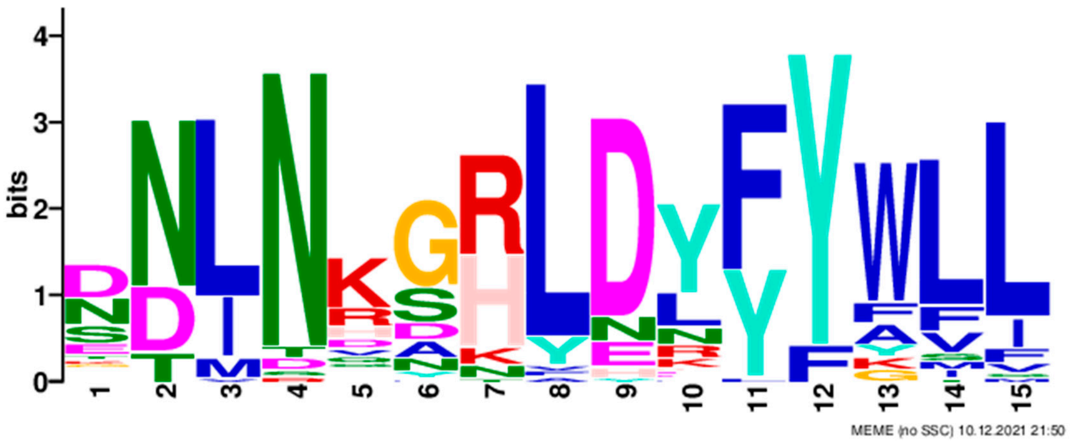

Motif 7

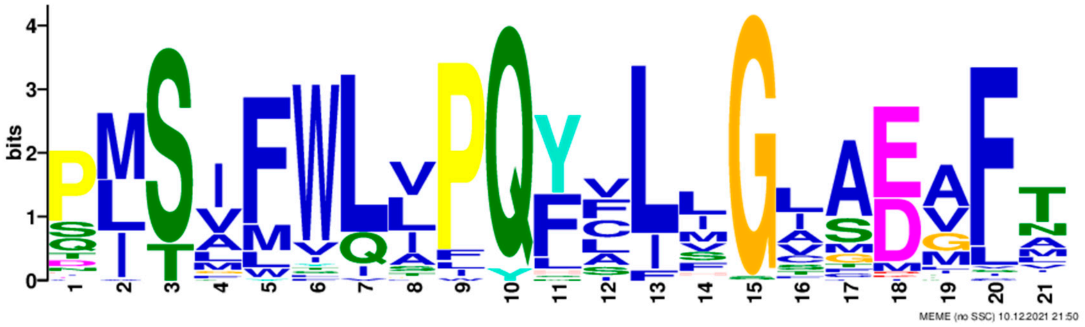

Motif 8

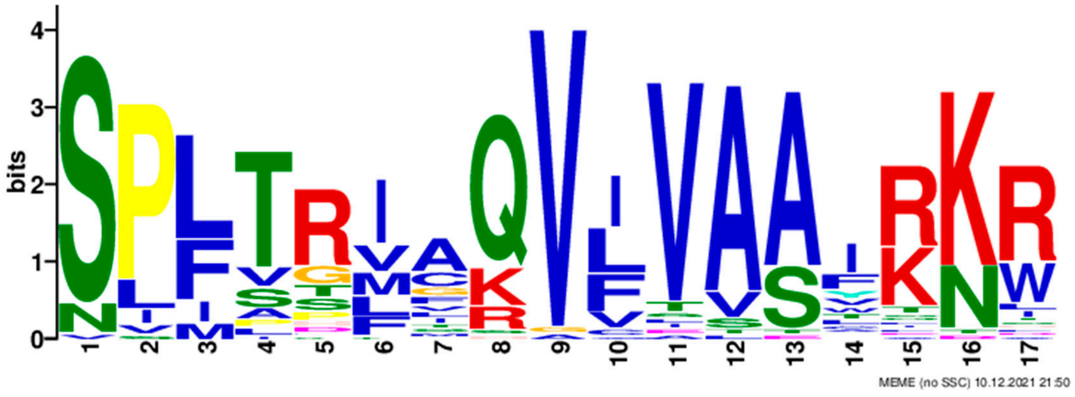

Motif 9

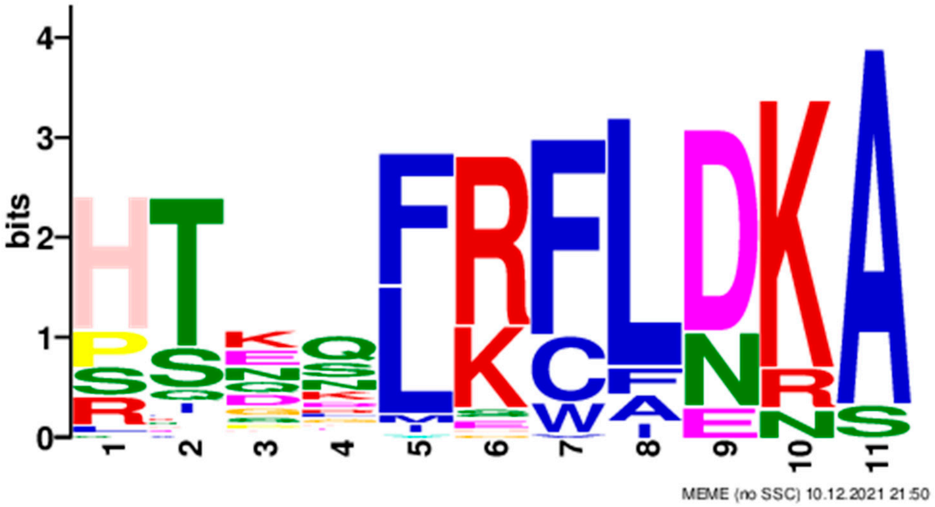

Motif 10

Figure S1. Detail information about motif sequences

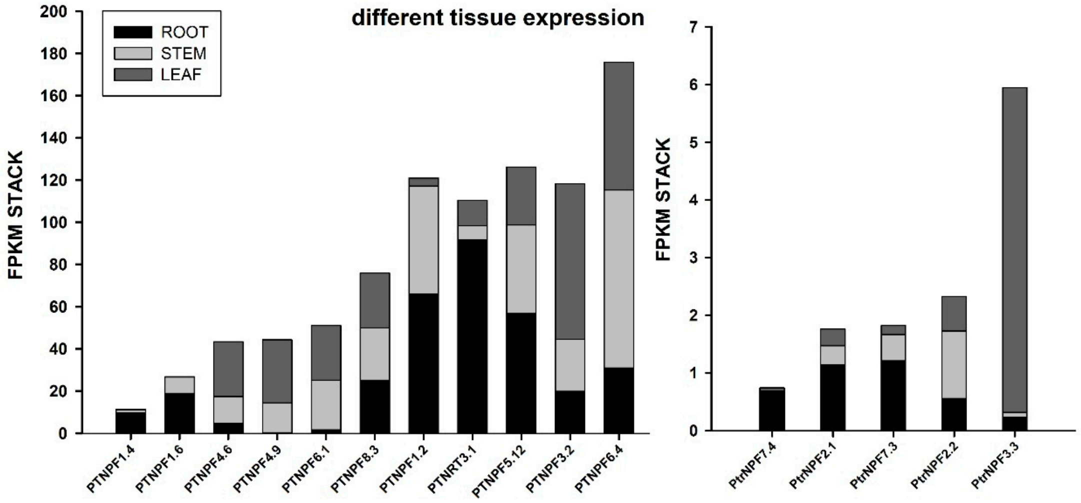

Figure S2. FPKM stack of differential tissue expression

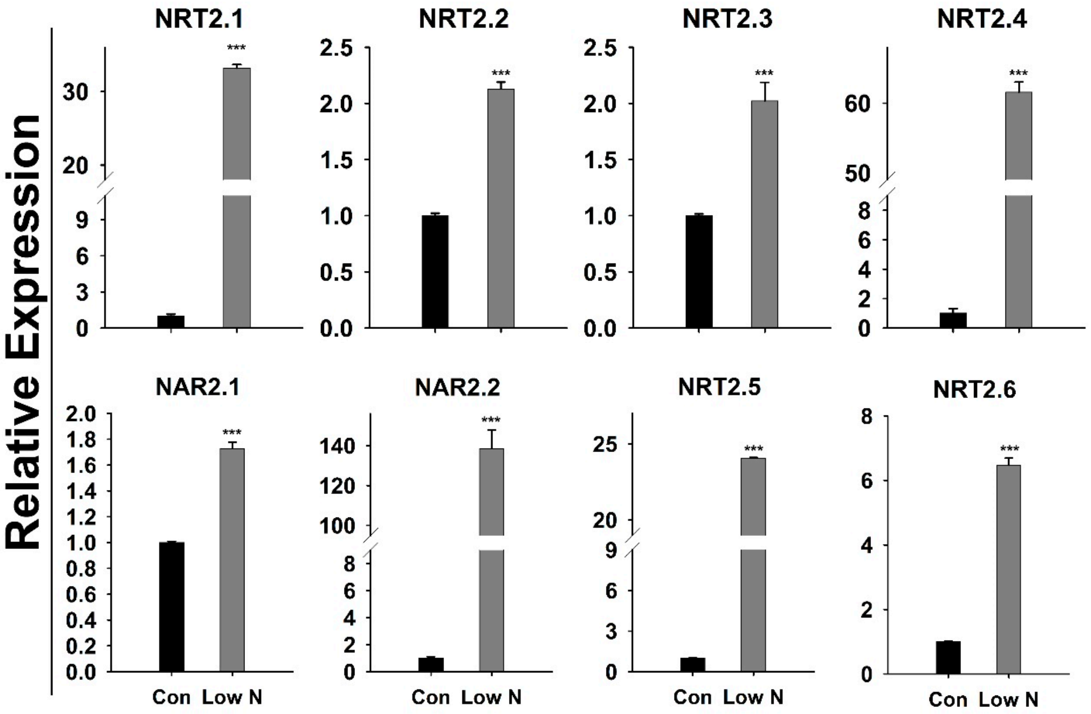

Figure S3. The relative expression of selected *PtrNRT* members under the low nitrogen condition. * Represented significant differences in comparison with control group using Student's t test at $0.01 < P < 0.05$. ** Represented significant differences at $0.001 < P < 0.01$. *** Represented significant differences at $P < 0.001$.

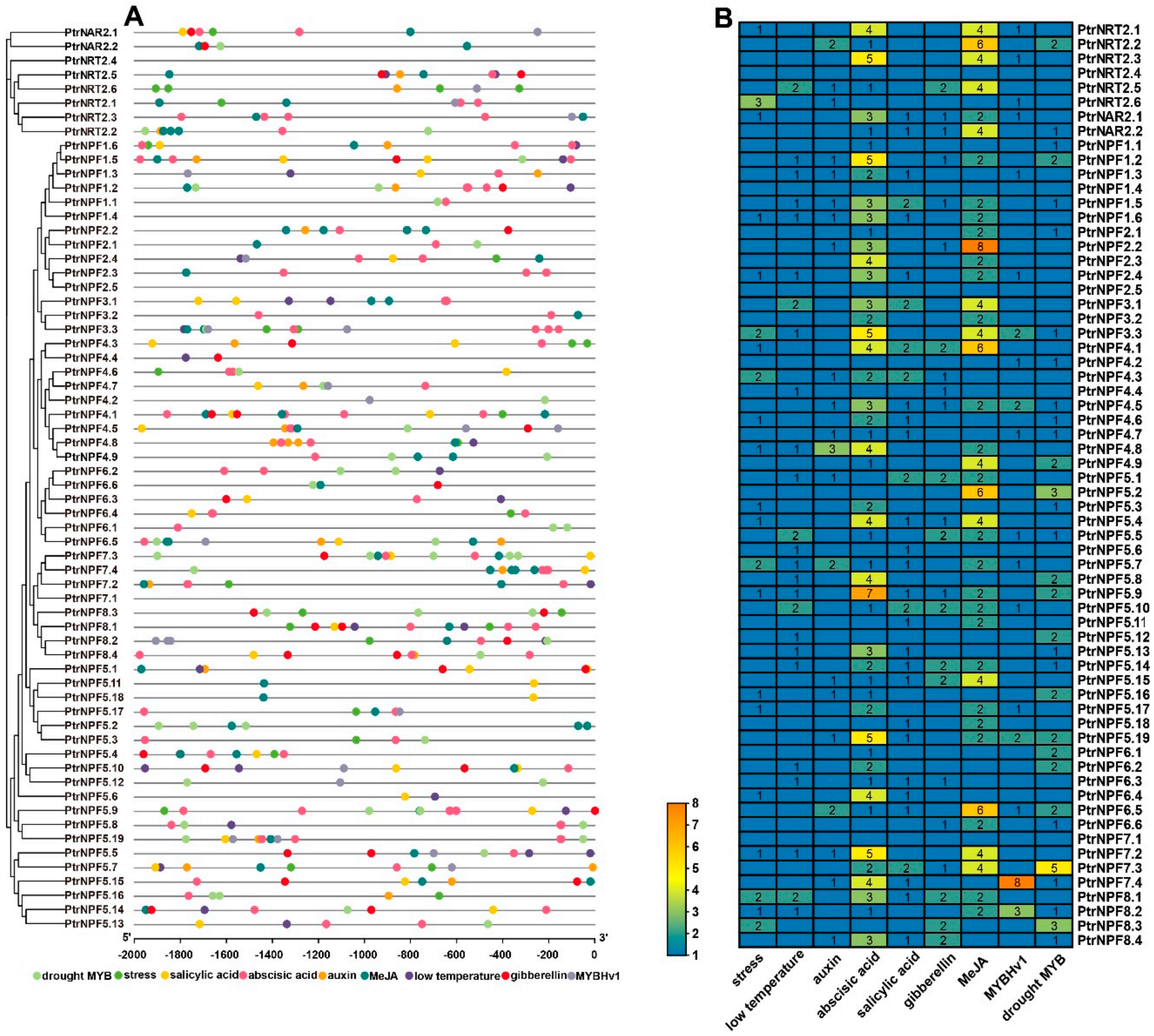

Figure S4. ***Cis*-acting element analysis of *PtrNRT* promoters**. (**A**) The position of *cis*-acting elements in *PtrNRT* promoters. The promoters were both uniformly extract 2000 nucleotides from upstream of gene coding sequences. (**B**) A heat map contained *cis*-acting elements occurrence frequency on the promoter of *PtrNRT* genes. The number represented the element frequency of occurrence. The upstream 2000 nucleotide sequences upstream coding sequence were analysis by the PlantCARE (http://bioinformatics.psb.ugent.be/webtools/plantcare/html/). Stress represented the *cis*-acting element involved in defense and stress response. Low temperature was the *cis*-acting element involved in low-temperature response. Auxin represented the auxin-responsive element. Abscisic acid represented the *cis*-acting element involved in the abscisic acid response. Salicylic acid represented the *cis*-acting element involved in salicylic acid response. Gibberellin represented the *cis*-acting element involved in gibberellin- responsive. MeJA represented the *cis*-acting regulatory element involved in the MeJA- responsive. MYBHv1 represented the MYBHv1 binding site. Drought MYB represented the MYB binding site involved in drought- responsive.

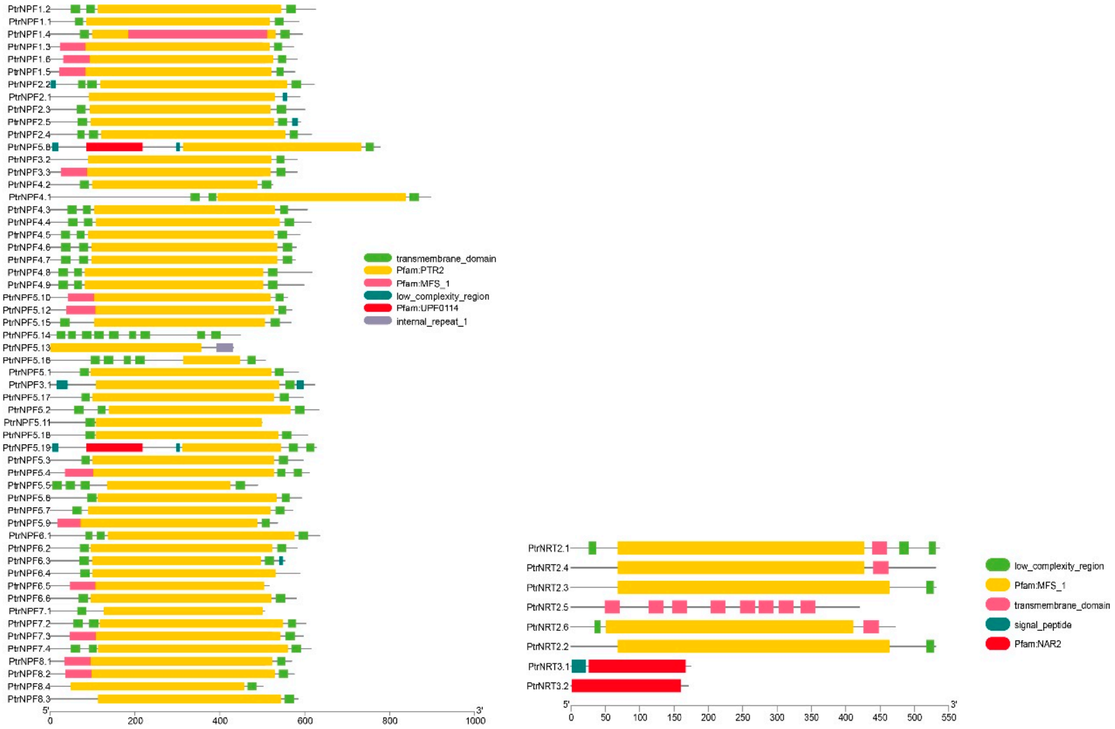

Figure S5. Smart domain visualization
